# The extra-thyroidal distribution of sodium iodide symporter

**DOI:** 10.3389/fendo.2025.1567405

**Published:** 2025-07-03

**Authors:** Rinae Prisca Gadisi, Meleshni Naicker, Strinivasen Naidoo

**Affiliations:** ^1^ Department of Therapeutics and Medicine Management, College of Health Sciences, University of KwaZulu-Natal, Durban, South Africa; ^2^ Department of Human Physiology, College of Health Sciences, University of KwaZulu-Natal, Durban, South Africa

**Keywords:** sodium-iodide symporter, thyroid-specific protein, extra-thyroidal expression, cellular and molecular distribution, cancer

## Abstract

The intrinsic transmembrane protein known as sodium iodide symporter (NIS) facilitates the active transport of iodide across the basolateral membrane of thyroid follicular cells. Iodine is an essential molecule that is used to produce the classical thyroid hormones that contribute to growth and development of various parts of the body and play a significant role as metabolic regulators. The cloning of the human NIS gene in 1996 has led to widespread advancements in thyroid-related research. Amongst these, the significant discovery of extra-thyroidal expression of thyroid-specific genes and proteins such as NIS in both human and non-human subjects has been well documented. The identification of NIS protein in non-thyroid tissue provides potential targets for therapeutic interventions aimed at modulating autoimmune responses in various parts of the body. In the diagnosis and prognosis of different types of cancer, the molecular and protein expression of NIS may prove to be an important cancer biomarker. This review will cover the extra-thyroidal distribution of NIS, as well as its pathophysiological implications in various tissues of human and non-human organs.

## 1 Introduction

### 1.1 Sodium-iodide symporter

Iodine is an essential molecule in the synthesis of thyroid hormones (THs), which play a crucial role in the growth and development of the central nervous system (CNS), musculoskeletal system, and respiratory system, and are also considered important metabolic regulators (1, 2). Iodide (I^-^) is actively co-transported into the thyroid gland via sodium iodide symporter (NIS). NIS is an intrinsic plasma membrane protein that is situated at the basolateral surface of the thyroid follicular cells (3). Cloning of the human NIS gene in 1996 has led to significant advancements in thyroid research and has resulted in molecular and protein characterization of NIS (4).

In 1996, rat NIS was cloned and sequenced from the Fisher rat thyroid cell line (FRTL-5)-derived cDNA library, revealing a 618 amino acid sequence (4) that is 87% homologous to the later cloned human NIS (5). Subsequently, the mouse gene was cloned from thyroid cDNA with an open reading frame of 1857 nucleotides that codes for a protein comprised of 618 amino acids, denoting 95% similarity to rat NIS and 84% similarity to human NIS (6). Human NIS is encoded by the NIS gene, SLC5A5 (4, 5). The human NIS gene is positioned on chromosome 19p12-13.2, with an open-reading frame of 1929 nucleotides coding a glycoprotein of 643 amino acids relative to a molecular mass of 70–90 kDa, attributed to inconsistent post-translational modification (glycosylation levels) (7). Ravera and colleagues (2022) were the first to report on the structure of rat NIS protein by using a single-particle cryo-electron microscopy, at an overall high resolution of 3.46 Å, establishing the basis for comprehending how NIS functions as well as its transport mechanism (2).

### 1.2 Role of NIS in thyroid hormone synthesis

In 1896, Baumann first described the thyroid gland’s ability to accumulate I^-^ (8). Functionally, it has been discovered that NIS co-transports one I^-^ anion against its electrochemical gradient simultaneously with two sodium (Na^+^) cations along their electrochemical gradient across the basolateral membrane of thyroid cells (7). The resultant transmembrane Na^+^ gradient serves as the driving force for the transportation of I^-^, which is supplied by the ouabain-sensitive Na^+^K^+^-ATPase pump (3, 7). Iodide is then translocated across the basolateral membrane through the apical membrane into the thyroid colloid by the PDS gene product known as Pendrin, which is a chloride/iodide transporter (7). Thereafter, I^-^ organification occurs, which involves I^-^ covalent incorporation onto tyrosyl residues alongside the thyroglobulin (TG) backbone within the colloid (9). Iodide organification is catalyzed by thyroid peroxidase (TPO), which allows for the synthesis of the THs thyroxine (T_4_) and triiodothyronine (T_3_) via the coupling of monoiodotryrosine (MIT) and diiodotyrosine (DIT) residues. Thyroid hormones are then stored in the colloid until they are stimulated by the release of thyroid stimulating hormone (TSH) into the bloodstream (10). Thyroid hormone synthesis can be interrupted by perchlorate and thiocyanate, which are NIS substrate analogues that act as competitive inhibitors of iodide uptake in the thyroid (11). At 5µM concentrations or less, perchlorate suppresses I^-^ transport, by changing the stoichiometry of wild-type NIS mediated I^-^ transport from electrogenic to electroneutral, which significantly downregulates the transmembrane Na^+^ gradient (12). In addition, the binding of perchlorate at the allosteric site restricts Na^+^ from binding to one of its two Na^+^ binding sites. Research has shown that perchlorate has an impact on sexual development by engaging with SLC5A5 in reproductive tissues (13).

## 2 Evidence for the extra-thyroidal distribution of NIS in human and non-human tissue

Despite NIS being primarily associated with the thyroid gland, widespread extra-thyroidal localization of active I^-^ transport has been documented; indeed, NIS has been identified in the placenta, ovary, testicular cells, pancreas, lacrimal gland, Gastrointestinal (GIT) tract, salivary glands, kidney, breast, lung, and liver (**Table 1**) (14–35),. Using a whole-body PET imaging and OLINDA/EXM software, Marti-Climent et al. (2015) evaluated the biodistribution of [^18^F]-tetrafluoroborate and calculated the absorbed doses in cynomolgus monkeys following intravenous administration of [^18^F]-tetrafluoroborate (36). The authors successfully visualized various organs at different time intervals, throughout the 132-minute imaging period, with an effective dose of 0.025mSv/MBq. The biodistribution pattern of the radiotracer mimics the physiological localization of NIS in the thyroid, urinary bladder wall and stomach wall.

**Table 1:** Summary of the extra-thyroidal tissue distribution of NIS.

Tissue	Species	Protein	mRNA	Methodology	References
Pancreas	human	+	NR	IHC	(14)
Lacrimal gland	human	+	+	RT-PCR, IHC	(14, 15)
Gastrointestinal tract	Human, rat, mice	+	+	IHC, Immunoblot assay, RT-PCR, *in vivo* intestinal uptake	(14, 16, 17)
Salivary glands	human	+	NR	IHC	(14, 18)
Kidney	human	+	+	IHC, RPA, RT-PCR, Western blot, Southern hybridization	(19, 20)
Placenta	human	+	+	IHC, RT-PCR	(21–23)
Breast	human, mice	+	+	RTq-PCR, immunoblot assay	(24, 25)
Testicular cells	human, mouse, rat	+	+	IHC, Western blot, RTq-PCR	(20, 26, 27)
Ovary	human, mice	+	NR	IHC	(28)
Lung	Human	+	+	IHC, RT-PCR, Western blot	(29–32)
Liver	Human, rat	+	+	IHC, RTq-PCR	(33–35)

NR, not reported; IHC, immunohistochemistry; RPA, ribonuclease protection assay; RTq-PCR, reverse transcriptase quantitative polymerase chain reaction; RT-PCR, reverse transcriptase polymerase chain reaction; +, present.

Although NIS is expressed in extra-thyroidal tissues, its expression levels are not sufficient enough to contribute to iodide homeostasis, implying that only the NIS gene in the thyroid gland is primarily responsible for maintaining iodide balance (37). In the context of human disease, the most significant findings to date are that the changes in the expression levels of proteins involved in iodine metabolism are implicated in tumor growth and play an essential role in cancer pathogenesis. However, the nature of this association can differ (38). In this review, we discuss the distribution and cellular expression of NIS in various human and non-human tissues, as well as the potential effects that the localized thyroid protein facilitates within these tissues.

### 2.1 Immunoreactivity of NIS in human exocrine glands

In a study by Spitzweg and colleagues (1999), those authors provided an analysis of NIS protein in several human exocrine glands (14). Exocrine glands consist of secretory segments, acinar cells that specialize in the secretion process, and ductal cells that facilitate ion transport via ion channels and transporters and move secretions to the epithelial surface. That study used immunochemical staining, along with monoclonal and polyclonal antibodies directed against human NIS protein, to evaluate its distribution in tissue segments of healthy human lacrimal and salivary glands, colonic, gastric and pancreatic mucosa. Notably, in the lacrimal gland, protein expression of human NIS was demonstrated to be concentrated at the basolateral membrane of the interlobular ductal cells and less concentrated in acinar cells. Those authors further observed similar findings in human salivary gland where NIS was more readily detected in ductal cells than in acinar cells. In addition, in colonic mucosa, NIS protein expression was limited to epithelial cells lining the mucosal crypts; in gastric mucosa, NIS protein was confined to parietal cells and gastric chief cells, and lastly, the pancreas demonstrated strong NIS protein expression in islet cells despite being weakly expressed in parenchymal cells and ductal cells. According to Spitzweg et al. (14) and Markitziu et al. (39), it is likely that human NIS plays a role in both the pathophysiology of the salivary and lacrimal gland dysfunction following radioiodine therapy and in the active iodine transport in these exocrine glands’ ducts. In fact, Spitzweg et al. (14) further suggested that the expression of human NIS protein in gastric and colonic mucosa may even mediate I^-^ transport in the gastrointestinal tract (GIT). Majerus and Courtois (40) reported that inorganic I^-^ is secreted by exocrine glands and is followed by the production of hypoiodite by oxidation, which can function as an antibacterial agent, thereby providing mucosal defense against environmental microbes. Further, Spitzweg et al. (14) concluded that I^-^ organification is a necessary requirement in these non-thyroidal tissues and suggested that future research should investigate the immunological significance of human NIS protein expression in human exocrine glands.

### 2.2 NIS gene and protein expressed in lacrimal sac tissue

In a later study, Morgenstern and colleagues (2005) further assessed the active transport of I^-^ in the lacrimal system of fresh human tissue samples using immunohistochemistry and reverse transcriptase polymerase chain reaction (RT-PCR) (15). Those RT-PCR results demonstrated NIS cDNA fragments measured 377bp in length and proved that NIS mRNA is expressed in normal lacrimal sac tissues. Similarly, their results from immunohistochemical staining demonstrated that NIS protein is expressed at the basolateral membrane of stratified columnar epithelial cells in nasolacrimal duct and lacrimal sac. NIS protein could not be detected in nasal mucosa, conjunctiva, Wolfring and Krause glands and lacrimal glands. This brings into contrast the contradictory findings of the older Spitzweg study (14), which reported NIS protein expression in the lacrimal gland. Technically, it is possible that the sensitivity of the immunohistochemical analysis was influenced by using both monoclonal and polyclonal antibodies in the earlier study, whereas the latter Morgenstern study (15) had only used a monoclonal antibody. Morgenstern and colleagues (15) had implied that the expression of NIS in the lacrimal sac may permit iodine to be recycled. They posited a mechanism whereby iodine is secreted by NIS into the lumen of the lacrimal sac; thereafter, it travels to the nasopharynx and digestive tract where it is easily absorbed through the small intestine.

### 2.3 Immuno-localized NIS protein in GIT tract

Using immunohistochemical and immunoblot analyses, Altorjay and co-workers (2007) investigated NIS protein expression in normal and diseased human gastrointestinal tissue (16). Those authors immuno-localized NIS protein in normal gastric mucosa, specifically in the basolateral region of gastric mucosal epithelial cells. In addition, they could not detect NIS protein expression within normal parietal cells, gastric chief cells and foveola cells. These negative findings of NIS protein expression in gastric mucosal parietal and chief cells appear to contradict earlier observations by the Spitzweg group (14). Possible explanations for these disparate observations may be that functional NIS protein expression is focal rather than widely sequestered and that differences in distribution may manifest due to fixation procedures that result in dissimilar tissue antigenicity (16). Altorjay and co-workers (2007) reported no NIS immunodetection in Paneth, goblet, and ciliated epithelial cells of the gastric intestinal metaplasia nor the goblet cells of the colon and squamous esophageal epithelium (16). In gastric tumor tissue, using immunoblot assays, those authors reported that 71% of their samples did not express NIS protein whilst the remaining 29% showed a significant reduction in NIS expression. Further, their immunohistochemical analyses demonstrated no NIS protein expression in gastric cancer, irrespective of papillary, signet-ring cell or adenocarcinoma subtypes. NIS expression was also absent in phenotypically normal tissues adjacent to the tumor margins in more than half of the cases examined whilst weak focal expression was observed in remaining cases. The characteristic linear plasma membrane NIS protein expression pattern was only detected significantly peripheral to the tumor. Thus, it is postulated that NIS could be a primary tumor marker in the diagnosis and prognosis of cancerous gastroesophageal lesions. In a later study by Nicola et al. (2009), NIS mRNA was shown to be expressed along the villus-crypt axis of the small intestine in rats by RT-PCR; NIS protein was expressed in the apical surface of enterocytes in rats and mice by immunohistochemistry (17). Additional studies were carried out by Nicola et al. (17), to determine whether I^-^ absorption is mediated by NIS in rat enterocytes: the authors performed an *in vivo* intestinal uptake. Firstly, they administered pertechnetate at 2-4h intervals, then both pertechnetate and perchlorate via duodenal catheter. Thereafter, blood samples were collected. The results indicated that NIS is significantly and presumably the core element of I^-^ accumulation in small intestines, playing a vital role in thyroid hormones synthesis.

### 2.4 NIS-mediated iodide uptake in human salivary tissue

In a separate study, and to better understand the modulation of NIS in salivary glands, La Perle and co-workers (2013) used immunohistochemical analyses to evaluate NIS protein expression in human salivary gland tissue (18). Those authors acquired normal, sialadenitis (inflamed), and neoplastic salivary tissues. In normal salivary tissue, NIS protein immunostaining was prominent along the basolateral membrane of striated duct cells, whilst weakly expressed in intercalated and excretory duct cells. In contrast, acinar cells of normal salivary tissue did not demonstrate positive NIS protein immuno-staining. Those findings of positive NIS expression in salivary ductal cells and its absence in acinar cells correlate well with earlier observations made by Spitzweg and co-workers (14). Additional findings in normal salivary tissue by the La Perle group (18) included increased NIS-positive expression of striated duct cells in submandibular glands compared to minor and parotid salivary glands. Following investigations in diseased salivary gland tissue, those authors reported reduced NIS expression in both inflamed and neoplastic human salivary glands. More specifically, NIS expression in striated salivary ducts was reduced in inflamed salivary glands compared to normal salivary tissue. In those striated duct cells undergoing goblet cell metaplasia, NIS reduction was more noticeable; whereas in lymphoepithelial lesions it was absent. Further, reduced NIS expression in striated ducts was observed in sialadenitis salivary tissue. In neoplastic human salivary glands, those authors demonstrated an absence of NIS expression in most benign and malignant neoplasms of ductal origin. The benign tumors examined were canalicular adenoma, papillary oncolytic cystadenoma, monomorphic adenoma, Warthin’s tumor, and pleomorphic adenoma. In Warthin’s tumor, NIS expression corresponded with a 2+ to 3+ staining intensity in many tumor cells whilst a few papillary oncolytic cystadenomas showed some evidence of NIS expression when assigned with a 1+ staining intensity in the minority of tumor cells. Evaluations of such malignant neoplasms as polymorphous low-grade adenocarcinoma, mucoepidermoid carcinoma, carcinoma ex pleomorphic adenoma, ductal carcinoma and adenoid cystic carcinoma were mostly negative for NIS expression. La Perle et al. (18) suggested that their findings of reduced NIS protein expression in inflamed salivary striated ducts would prove useful in developing novel strategies to prevent or reduce salivary gland damage caused by the effects of radioactive iodine treatment in thyroid cancer patients. They also suggested that subsequent research should identify pharmacological agents that may enable the selective inhibition of NIS activity in salivary glands to patients receiving radioactive therapy.

### 2.5 Renal tubular cells mediated by NIS

The urinary excretion of I^-^ is thought to occur through a combination of glomerular filtration, partial tubular reabsorption and secretion of plasma I^-^; however, the exact transport mechanisms of I^-^ in the renal tubular system have remained largely unknown (41). Since NIS is an essential protein that mediates the transport of I^-^ in the thyroid gland as well as in other extra-thyroidal tissues, it is likely that I^-^ transport in renal tubular cells is also mediated by NIS. This rationale was the basis for further studies by Spitzweg et al. (2001), where NIS mRNA and protein expression was demonstrated within normal human kidney tissues using RT-PCR, Southern-blot hybridization, ribonuclease protection assay (RPA), Western-blot and immunohistochemistry analysis (19). Automated sequencing of the resulting amplificants revealed complete congruity to the published human thyroid NIS cDNA sequence (5). Full-length human NIS mRNA expression was detected in normal human kidney tissue by RT-PCR followed by Southern analysis. The RPA assays demonstrated positive protected bands at 483 bp of human NIS mRNA. Western blotting revealed the presence of the human NIS protein in human kidney cells measured ~80kDa. Immuno-localisation of NIS was detected prominently at the basolateral region of proximal tubular cells, and in distal tubular cells appeared more diffuse within the cytoplasm, whilst NIS protein was undetected in the glomeruli. Subsequently, Wapnir et al. (2003), utilizing tissue microarray cores from normal human kidney (20), demonstrated staining at the intercalated and apical surface of principal cells of renal distal and collecting tubules. This evidence of functional human NIS expression in distinct tissues of the nephron suggests that renal I^-^ transport could be, at least partially, an active process regulated by NIS (19).

### 2.6 NIS immuno-reactivity in placental villous tissue

In comparison to human thyroid tissue, low levels of NIS and Pendrin (PDS) were detected in human placental villous tissues, acquired at the early and late gestational periods, using

RT-PCR (21). Pendrin is a transmembrane transporter protein expressed by the Pendred syndrome gene (*SLC26A4* PDS) and it facilitates the transport of I^-^ into the thyroid colloid (42). In the Bidart study (2000), immunohistochemical analysis revealed that NIS protein was expressed on the membrane of villous cytotrophoblast cells, whereas PDS was particularly expressed at the brush border membrane of villous syncytiotrophoblast cells facing the maternal blood (21). Since the placenta acts as a barrier between the maternal and fetal compartments and is almost impermeable to the mother, it is crucial to the functioning of the fetal thyroid system. Thus, those investigators suggested that future studies should aim to determine the consequences of pathophysiological dysregulation of I^-^ during pregnancy. It is those findings of low NIS protein expression in human placental tissue by the Bidart group (2000) that prompted later work by Di Cosmo et al. (2006) where immunohistochemistry was used to determine NIS protein expression in human placenta obtained during the first trimester and at full term of pregnancy (22). Those results demonstrated NIS immunoreactivity in cyto-syncytiotrophoblast cells, in mesenchymal and endothelial cells, in decidual cells, and in the endometrial glands. Interestingly, those authors noted that whilst the placenta is larger in size than the thyroid gland, it does, however, contain lower levels of NIS protein expression in comparison. They also concluded that protein expression of NIS is thought to be continual throughout the gestational period since NIS was observed in placental villous tissues of early and late gestational period (22). Trovato et al. (2008) reported the expression of NIS protein on the endometrial mucosa of sterile female, as was demonstrated by immunohistochemistry (23). The authors indicated that NIS expression may serve as a new putative biomarker for female sterility. A recent study by Sun et al. (2021), demonstrated that the expression of iodide transporter Pendrin is upregulated during iodine deficiency to supply iodine and inhibit maternal iodine deficiency from affecting the fetus (43). Further exploration was carried out to determine the differences in iodine metabolism in pregnant rats. The rats were categorized into four groups: low iodine, normal iodine, tenfold high iodine and fiftyfold high iodine (44). Iodine intake and excretion were calculated from the 15^th^ day of pregnancy, after one week of adaptive feeding. Iodine concentrations in placenta, thyroid, fecal and urine were measured by Inductively Coupled Plasma Mass Spectrometry (ICP-MS). Those authors noticed that iodine storage and excretion in the thyroid gland and placenta are positively proportional with iodine intake.

### 2.7 NIS profiling in normal, fibroadenoma, and cancerous breast tissues

In a previous study by Tazebay et al. (2001), those authors used an immunoblot assay to detect NIS protein in mice mammary gland tissue (24). Those investigators administered oxytocin, prolactin, and estrogen (in the absence of progesterone) to ovariectomized mice. Interestingly, high levels of mammary gland NIS expression were observed in ovariectomized mice despite the administered hormone combination closely simulating their relative levels in lactating mice. Ryan and co-workers (2011) investigated the expression of NIS mRNA and its potential effect in human breast tissue (25). Human breast tissue specimens (normal, fibroadenoma, and malignant) were analyzed by reverse transcriptase quantitative polymerase chain reaction (RTq-PCR). Their results revealed low levels of NIS mRNA in normal breast tissue whilst, in contrast, significantly higher levels were observed in malignant tissue with fibroadenoma being the highest. Thus, that study conceded that NIS mRNA expression in breast cancer is not a reliable indicator of malignancy. According to Ryan and colleagues (25), their data demonstrated that the expression of NIS in mammary gland anticipates suckling, and that lactation is vital for newborns because maternal milk is their only source of I^-^.

### 2.8 NIS detection in testicular tissue

Previous studies have demonstrated NIS expression in testicular tissue, but did not specify the exact intracellular localization of NIS (20, 26). Russo and co-workers (2011) investigated the expression and localization of human NIS mRNA and protein, respectively, in normal testicular tissue (27). That group had rationalized that the expression of NIS is a pre-requisite in predicting radioiodine accumulation in the gonads of male thyroid cancer patients receiving such treatment. They further noted a lack of research on the molecular processes underpinning radioiodine concentration in testes. Thus, in their study, they analyzed the expression of NIS mRNA and protein in both fetal and adult mouse, rat, and human normal testicular tissue, utilizing RTq-PCR, immunohistochemistry and Western blot. Their RTq-PCR data revealed the expression of NIS mRNA in fetal and adult human and mouse testes, albeit a greater expression in adult testes was detected than in fetal testes. Their immunohistochemical analyses demonstrated abundant positive NIS protein immunostaining in cells adjacent to the lumen of seminiferous tubules in fetal and adult testicular tissues for all groups. Further immunohistochemical analyses demonstrated NIS protein expression in Leydig cells, specifically in the interstitial space surrounding the seminiferous tubules. They observed no positive NIS protein expression in Sertoli cells within the mouse, rat, and human testicular tissue. In testicular tissue obtained from a patient suffering from spermatid maturation arrest, a syndrome marked by inadequate spermatogenesis development, NIS staining was primarily detected in the cytoplasm and faintly in the plasma membrane of Leydig cells but not in germ cells. Further, investigations included the extraction of protein from human adult normal testicles and neoplastic thyroid tissues, that were subjected to Western blot analysis to identify for NIS protein expression. Human testicular tissue protein extracts revealed a distinct band, approximately 80 kDa, corresponding to the human NIS protein. However, this band was less intense than that of neoplastic thyroid tissue extracts. According to their results, those investigators suggested that the expression of NIS may serve as a molecular basis for radioiodine uptake in testicular cells. Clinically, this would account for the observed impairments in gonads of adult male patients receiving radioiodine treatment for thyroid cancer.

### 2.9 NIS protein presence in ovary and ovarian tumor cells

In a study conducted by Riesco-Eizaguirre et al. (28), NIS expression was examined in normal ovary and ovarian tumor tissue. Immunohistochemistry on paraffin-embedded ovarian tissue derived from the ovaries of fourteen women demonstrated no histological changes, excluding three female patients who had inclusion cysts. Further, immuno-localized NIS protein was observed on the basolateral membrane of ovarian surface epithelium, fimbriae cells and the fallopian tubal epithelial cells. The fallopian tubular epithelial cells comprise ciliated and secretory cells, where secretory cells demonstrated stronger NIS expression than ciliated cells. In contrast, stromal cells of the ovary did not show any evidence of NIS protein immunostaining. Additional findings include those three patients presenting with inclusion cysts where NIS protein was expressed in cyst epithelia. In that same study, the authors further explored NIS protein expression in ovarian cancer: Two spontaneous ovarian tumor samples from a transgenic mice model demonstrated NIS overexpression in tumor cells, particularly intracellularly and at the plasma membrane. It was suggested that the expression of NIS could present as an ovarian cancer marker. Further, and perhaps more significantly, it has been postulated that ovarian NIS could allow for the use of radioiodine in the diagnosis and treatment of ovarian cancer. It is crucial to mention that enhancing the oxidation of iodide within tumor cells may upregulate iodide’s retention and improve the effectiveness of radioiodine (38).

### 2.10 Expression of NIS protein in primary lung cancer

Fragoso and colleagues (2004) investigated whether thiocyanate is transported via active mechanisms of the airway epithelia (29). Immunohistochemistry demonstrated NIS protein expression in submucosal glands and basal surface of gland acinar cells in human trachea. In Ali cultures of the human airway epithelia, immunolocalized NIS protein was also detected in half of the cells. NIS mRNA was shown to be expressed in the airway epithelial cells using RT-PCR. The epithelia secrete thiocyanate, providing a defense mechanism to regulate peroxidase-mediated host defense activity on the apical surface. Those authors established that NIS may be a vital component in transporting and concentrating thiocyanate into airway cells, and that cystic fibrosis transmembrane conductance regulator (CFTR) modulates the transport into the airway channel. Lee et al. (2006) suggested that radioiodine could potentially be used for the diagnoses and treatment of lung adenocarcinoma (30), particularly for the treatment of patients with lung adenocarcinoma and deficient glucose transporter 1 protein (31). Interestingly, this study further showed NIS protein expression in 75 of 139 cases of lung adenocarcinoma using immunohistochemistry (30), and validation by western blot (31). In a more recent study, Lu and colleagues (2021) detected significant levels of NIS expression in one primary lung cancer, which demonstrated the uptake of Radioactive Iodine (RAI) avidity, compared to seven lung cancers with lower levels of NIS expression and incapable of exhibiting RAI avidity (32). According to those authors, their findings are crucial for clinicians to be mindful of when interpreting RAI scintigraphy.

### 2.11 NIS protein found in the liver metastases

The functionality of NIS in human liver cancer, and in diethylnitrosamine (DEN)-induced Wister rat model of primary liver cancer at different stages of carcinogenesis, was investigated by Liu and co-workers (33). Both NIS mRNA and protein expression was confined to cholangiocytes in human cholangiocarcinoma (CCA), but not in human hepatocellular carcinoma (HCC) by RTq-PCR and immunohistochemistry respectively. In the DEN-induced rat model, the authors reported that NIS expression becomes prominent at the early stages of liver carcinogenesis. Additional findings in this study demonstrated enhanced NIS expression throughout monoclonal tumor growth; this enabled tumor to receive efficient RAI therapy. In a later study by Lacoste et al. (2012), NIS was shown to be expressed and mis-localized in liver metastases. Its expression is further associated with leukemia-associated RhoA guanine exchange factor (LARGE) that stimulates Rho to promote tumor cell metastases (34). Thereafter, an investigation by Guerrieri and colleagues (2013) determined the transcription factors and signaling pathways that regulate NIS expression in CCA, HCC and primary human hepatocytes (PHH) (35). In that same study, *in silico* analysis indicated that the proximal promoter SLC5A5 comprises of clusters of p53-responsive elements that enhance NIS expression in liver cancer cells. Doxorubicin stimulates NIS protein and mRNA expression in CCA and HCC, but not in PHH, inducing cancer cell death. The authors suggest that a combination of radioiodine therapy and doxorubicin would target NIS-expressing extra-thyroidal cancers, in this case liver cancer.

## 3 Conclusion

This mini review is intended to document the widespread extra-thyroidal expression of NIS, along with scientific interpretation and clinical inference of localized NIS in various tissues of human and non-human subjects. According to the numerous study data published thus far, a rational case could be made that NIS serves as a potentially significant biomarker in the diagnosis and prognosis of various malignancies. This suggests that thyroid hormones, long seen as physiological regulators crucial to metabolic processes, growth, and development, may have clinical relevance beyond thyroid disease. Further, identifying extra-thyroidal NIS expression as well as that of other thyroid-specific proteins suggests an implicit significance in the management of autoimmune thyroid disease and its associated complications. In a previous study conducted by our research group, we identified the neuro-localization of other thyroid-specific proteins, viz., thyroid stimulating hormone receptor and thyroglobulin in various areas of the normal adult human brain (45, 46), and from those studies have evolved our future endeavors to evaluate NIS protein expression in the human CNS. Findings of brain-derived NIS protein that may co-express with other thyroid synthesizing proteins could very well provide fresh perspectives on the neurological symptoms associated with autoimmune thyroid disease. The advancement of thyroid research, specifically related to extra-thyroidal expression, would contribute towards providing novel therapeutic approaches for several acute diseases and chronic malignancies.
